# Mid-Infrared Spectroscopy for the Qualitative and Quantitative Analysis of the Wheat Proteome

**DOI:** 10.1021/acs.analchem.5c05258

**Published:** 2026-01-21

**Authors:** Dedy L. Nadeak, Michael Wiederstein, Sabine Baumgartner, Elisabeth Reiter, Rudolf Krska, Stephan Freitag

**Affiliations:** a Department of Agricultural Sciences, Institute of Bioanalytics and Agro-Metabomics, BOKU University, 3430 Tulln an der Donau, Austria; b Austrian Agency for Health and Food Safety GmbH, Institute for Animal Nutrition and Feed, 1220 Vienna, Austria; c Institute for Global Food Security, School of Biological Sciences, Queen’s University Belfast, BT7 1NN Belfast, U.K.; d FFoQSI GmbH—Austrian Competence Centre for Feed and Food Quality, Safety and Innovation, 3430 Tulln an der Donau, Austria

## Abstract

Wheat contributes 19% of protein in the global human diet. Mid-infrared spectroscopy (MIR) combined with attenuated total reflection (ATR) is an excellent analytical method for assessing the chemical composition of complex biological samples. In contrast to established techniques used for wheat protein analysis, we show that by using ATR-MIR the protein secondary structures of different fractions can be studied. We found that albumin and globulin fractions were primarily composed of α-helix, with proportions of 57.8% and 45.9%, respectively. Gliadins, meanwhile, contained 38.3% β-turn and 36.9% α-helix, while glutenins predominantly exhibited 44.8% β-turn secondary structures. In addition, we found that by using analysis of variance (ANOVA) simultaneous component analysis (ASCA), the obtained MIR spectra of the wheat protein fractions were significantly (*p* < 0.001) affected by the sampling sites and variety. Quantification of the protein content of each sample through the amide II band also revealed significant differences (*p* < 0.001) across the sampling sites in the different protein fractions. The found concentration range of protein fractions within the wheat samples were as follows: 1.7–3.6, 0.4–2.4, 4.0–5.4, and 0.8–4.8 g/100 g of albumins, globulins, gliadins, and glutenins, respectively. In conclusion, this study shows that ATR-MIR has an immense potential for wheat proteome analysis.

## Graphical abstract

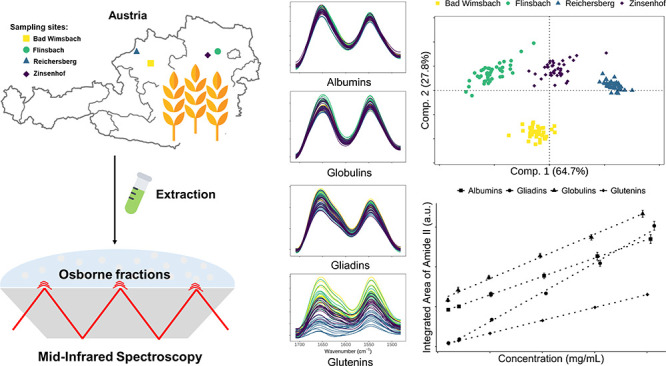

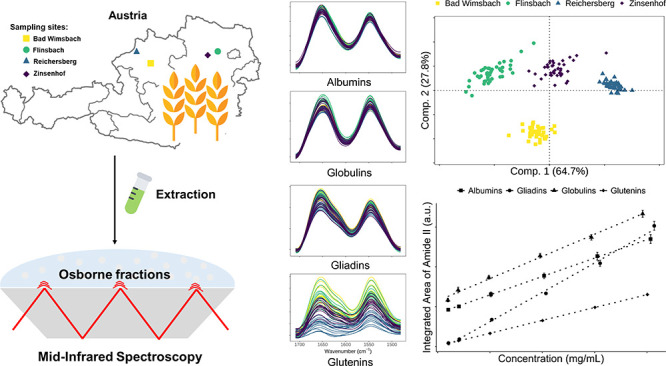

## Introduction

Wheat (*Triticum* L.) is one of the most widely consumed staple crops globally, with an estimated production in 2024 comprising 798.4 million tons and forecasted to grow to 871.7 million tons in 2033.^1^ Wheat grains typically contain between 7% and 20% of protein, depending mainly on its variety, growing environment and agronomical practices.^2^ The protein components in wheat play an important role for humanity, such as controlling the texture in baking, determining the processing properties in milling, and contributing to about 19% of the daily protein intake per capita worldwide.^2,3^ Wheat protein is commonly classified into the four Osborne fractions, albumins, globulins, gliadins, and glutenins, based on the different solubility of these groups of proteins.^4^ Studying these protein fractions is crucial for understanding viscoelastic properties in the food industry,^5,6^ in allergy and intolerance research,^7,8^ as well as in environmental and agronomic research.^9,10^

Protein analysis is available in a wide variety of methods. With qualitative methods such as sodium dodecyl sulfate–polyacrylamide gel electrophoresis (SDS–PAGE), the molecular weight of proteins can be studied.^12^ In addition, by combining SDS–PAGE with densitometry, a semiquantitative estimation of wheat proteins can be achieved.^13^ Simple quantitative methods based on colorimetry, the Lowry assay,^14^ the Bradford assay,^15^ and the bicinchoninic acid (BCA),^16^ are commonly used to determine protein concentrations. However, the use of coloring reagents as labels is error prone; thus, different assays obtain different values for the protein concentration of the same sample.^17^ Although strategies exist on selecting the appropriate assay,^18^ these dye-based methods cannot provide qualitative information. For a more sophisticated protein analysis, liquid chromatography (LC) coupled with ultraviolet (UV)^19^ spectroscopy and LC coupled with mass spectrometry (MS)^20–22^ are the two most commonly used methods. These methods can provide qualitative and quantitative information, including the concentration of proteins in each fraction, as well as their respective subtypes. For example, LC-MS can be used to identify certain peptides and reconstruct the original protein sequence. However, these methods are labor-intensive, time-consuming, and destructive; thus, they cannot be used for studying intact proteins and their secondary structures. Therefore, there is a need for analytical methods capable of obtaining quantitative and qualitative information about intact wheat proteins.

MIR has been employed to study the secondary structure of proteins using the amide I band (1700–1600 cm^–1^) but also for quantification using the amide II band (1600–1500 cm^–1^) in a nondestructive and label-free manner.^23–26^ Although MIR in combination with ATR is a promising technique for the quantitative and qualitative determination of protein content, it is still rarely used in food analysis.^27^ This might be linked to the fact that it is challenging to use MIR in an aqueous environment due to the overlap of the strong absorption of the O–H stretching vibration at 1640 cm^–1^ and the amide I band.^23,28^ Furthermore, the addition of non-infrared-active components, i.e., ions, alters the chemical environment which is in the end leading to erroneous spectra.^28^ However, such challenges might be overcome by selecting the right background, i.e., solvent, during sample analysis.

The objectives of this study were to use ATR-MIR to qualitatively and quantitatively analyze the Osborne fractions of wheat. Toward these objectives, an ATR-MIR calibration was developed based on in-house-made calibrants for albumin, globulin, gliadin, and glutenin fractions. Following the method development and validation, 60 Austrian wheat samples from four different sampling sites were analyzed. To the best of our knowledge, this is the first study to demonstrate the potential of ATR-MIR for quantitative and secondary structure analyses of wheat proteins.

## Materials and Methods

### Sample Collection

Samples were collected by the Austrian Agency for Health and Food Safety (AGES) in conjunction with the variety approval program, which aims to test traits in various crops.^29^ A total of 60 samples from 15 different varieties were collected in 2021. Four sampling sites were used to collect samples, namely, Bad Wimsbach (48°03′06.9″ N, 13°54′12.9′′ E), Flinsbach (48°14′20.8′′ N, 15°32′55.7′′ E), Reichersberg (48°19′55.8′′ N, 13°22′01.4″ E), and Zinsenhof (48°08′10.5″ N, 15°15′43.5″ E), with 15 samples (one sample per variety) collected per site. Each site conducted three or four replicate trials per wheat variety using a rectangular lattice design. Approximately 10 kg subsamples were collected from each of the four replicates, resulting in one sample for each variety and region. In the laboratory, a reduced composite sample weighing between 2 and 8 kg per variety was ground. The wheat samples were ground to 0.8 mm in size by using a Chopin grinder (KPM Analytics, Villeneuve-laGarenne, France). Subsequently, 200 g of each sample was analyzed by using ATR-MIR.

### Protein Extraction

Albumins and globulins were extracted according to the modified Osborne protocol of Lookhart and Bean.^4,30^ Albumins were extracted by adding 20 mL of deionized water (DW) to 2 g (±0.01 g) of ground sample in a 50 mL Falcon tube and mixing at 200 rpm for 30 min at 20 °C in an orbital shaker (IKA KS 4000, Staufen, Germany). The mixtures were then centrifuged at 4700 rpm at 20 °C for 5 min on an Allegra X-30R centrifuge (Beckman Coulter, Brea, United States). The supernatants were decanted into a new tube and stored as albumins. The remaining pellets were then extracted with 20 mL of 0.5 M NaCl in an orbital shaker at 200 rpm for 30 min at 20 °C followed by centrifugation (4700 rpm, 20 °C, 5 min) to obtain globulins. The pellets were vortexed with 20 mL of DW for 1 min and centrifuged, and the supernatants were discarded. This additional washing step was carried out in order to reduce the effect of the salt in the pellet on extracting gliadin in the following steps.^30^

The Dupont et al. method was used to extract gliadins and glutenins.^31^ To the remaining pellets, 30 mL of 0.25% (w/v) sodium dodecyl sulfate (SDS) in borate buffer (50 mM Na_2_B_4_O_7_·10H_2_O, pH 8.5) consisting of 0.1% (w/v) glycine was added, and the mixtures were shaken in an orbital shaker at 200 rpm for 1 h at 20 °C. Upon centrifugation (4700 rpm, 20 °C, 5 min), supernatants containing gliadins were transferred to new tubes. Following extraction of glutenins from the remaining pellets, 30 mL of 2% (w/v) SDS in 50 mM borate buffer (pH 8.5) consisting of 0.1% (w/v) glycine was added and shaken for 2 h at 60 °C, then cooled to room temperature (RT) and centrifugation at 4700 rpm (20 °C, 5 min). Three technical replications (separate extractions) were conducted for each sample.

### Protein Calibrants

Purified albumin, globulin, gliadin, and glutenin fractions to be used as calibrants were prepared based on the protocol reported by Schalk et al. with some modifications.^21^ For the calibrants, 100 g (±0.01 g) of ten different Austrian wheat cultivars (Erla Kolben, Ikarus, Arnold, RGT Reform, SU Habanero, Capo, Aurelius, Chevignon, Ethan, and Ernestus) were mixed and ground into a fine powder using a mill (Perten LabMill 3610, PerkinElmer, Springfield, IL, United States). Subsequently, ten-times 10 g (±0.01 g) of ground wheat mixture were defatted in 10 different 50 mL Falcon tubes by adding 25 mL of *n*-hexane/ethanol (95/5, v/v) to each tube for 30 min at RT, followed by centrifugation (3750*g*, 15 min, 20 °C). The obtained supernatants were discarded. Each pellet was extracted once more using 25 mL of *n*-hexane, centrifuged, and the supernatant was discarded. The pellets were placed under a fume hood for 5 days to completely remove the organic solvent. Following this, defatted flours were extracted three times with 20 mL of DW in an orbital shaker for 30 min at 20 °C. The suspensions were centrifuged (3750*g*, 20 min, 20 °C) and the supernatants were collected in new tubes and stored as albumins. The remaining pellets were processed using the same extraction workflow as for albumins but with different solvents for the other fractions, 0.5 M NaCl for globulins, 60% (v/v) ethanol for gliadins, and 50% (v/v) 1-propanol/0.1 M Tris-HCl (pH 7.5)/2 M urea containing 1% (w/v) dithiothreitol (DTT) for glutenins. The corresponding supernatants were combined and reduced in volume by evaporating them under vacuum (Hei-VAP Precision G3, Heidolph, Schwabach, Germany) to obtain approximately 100 mL of each protein fraction extract before dialysis. Afterward, the concentrated protein fractions were dialyzed with 6000 to 8000 molecular weight cutoff (Spectra/Por 1 RC, Repligen, Waltham, MA, United States) for 3 days against 0.01 M acetic acid and 1 day against DW.^32^ To obtain solid protein fractions, the dialyzed solutions were then lyophilized using a freeze-dryer (FreeZone6 Plus, Labconco, Kansas City, MO, United States). The protein content of each fraction was determined five times by Kjeldahl analysis (N × 5.7) on KjelLine (Buchi, Flawil, Switzerland) according to DIN 19 684–4. Protein contents of 64.5 ± 3.5% for albumins, 78.6 ± 8.2% for globulins, 76.9 ± 5.2% for gliadins, and 71.5 ± 3.0% for glutenins were found.

The differences in extraction efficiency between Schalk et al.^21^ for calibrant isolation and the protocol for protein fractions extraction by Dupont et al.,^31^ was studied. Therefore, the wheat calibrant material (10 ± 0.01 g) was also extracted using the protocol by Dupont et al., followed by dialysis and lyophilization. For solution preparation, 150 mg of gliadins and glutenins from calibrant and sample extraction techniques were dissolved in 10 mL of their respective extraction solution according to Dupont et al. All solutions, then, were measured six times using ATR-MIR with the corresponding background. Subsequently, normalization and baseline correction were performed to eliminate absorbance differences due to concentration prior to integration of the amide II band (see Figure S1). The correction factor (CF) was calculated as follows:

1 $$\mathrm{CF}=\frac{\mathrm{average of}A_{\mathrm{Schalk}}}{\mathrm{average of}A_{\mathrm{Dupont}}}$$

Correction factors of 0.886 and 0.985 were determined for gliadins and glutenins, respectively. These factors were used to correct protein concentration of the data according to the protocol of Dupont et al.

### ATR-MIR

An FTIR spectrometer (Invenio X, Bruker Optics, Ettlingen, Germany) equipped with a liquid nitrogen-cooled mercury cadmium telluride (MCT) detector and a triple-bounce diamond zinc selenide ATR element (MIRacle, PIKE Technologies, Madison, WI, United States) with a 45° angle of incidence was utilized for the spectroscopic measurements. For the measurement, 100 μL of the extract was pipetted onto the ATR crystal. The spectral acquisition was conducted by averaging 64 scans at a 4 cm^–1^ resolution. According to the Beer–Lambert law, absorbance is defined by the negative logarithm of transmittance, where *I*_0_ is the background and *I* the intensity after interaction with the sample. For the background measurements (*I*_0_), the respective solvents used for extracting the different protein fractions was used, while *I* was the obtained protein extract. The absorbance spectra of albumins, globulins, gliadins, and glutenins were obtained by measuring DW, 0.5 M NaCl, 0.25% SDS in borate buffer, and 2% SDS in borate buffer, respectively, as *I*_0_ with the corresponding extracts measured as *I*. The protein concentration had to fall in the calibrant range (0.5–10 mg/mL).

2 $$A=-\log\frac{I}{I_{0}}$$

Before spectra acquisition, 100% lines were recorded with a 60 s time difference. This measurement was repeated for five samples as a quality control measure. A root-mean-square noise in the 100% line smaller than 10^–5^ between 1700 and 1500 cm^–1^ was considered sufficient for this purpose. After collecting the spectra, the ATR crystal was thoroughly rinsed with ethanol and then with DW. All FTIR spectra, recorded using OPUS 8.7.31 (Bruker Optics, Ettlingen, Germany), were converted into a single comma-separated values (csv) format file using Spectragryph (Spectroscopy Ninja, Oberstdorf, Germany).

### Protein Quantification

The working range and the limit of quantification (LOQ) for different protein calibrations were determined following the Eurachem guide.^33^ For determining the working range, the protein calibrant was prepared at a concentration of 20 mg/mL, accounting for the purity of each protein fraction. The albumin, globulin, gliadin, and glutenin fractions were dissolved in DW, 0.5 M NaCl, gliadin, and glutenin extraction buffer, respectively. All stock solutions were then serially diluted to six levels of concentration (0.5–10 mg/mL), with two additional levels at 20% of the lowest (0.4 mg/mL) and the highest (12 mg/mL). All solutions were measured three times by ATR-MIR. To determine the LOQ value, ten replicate measurements of the extraction solution were carried out with itself as reference spectra according to the protein fractions. Subsequently, the LOQ was calculated using the following equation

3 $$\mathrm{LOQ}=10\times \frac{\mathrm{sd}}{\mathrm{slope}}$$

where sd is the residual standard deviation of the integrated amide II band of the corresponding protein extractant. The slope was obtained from the corresponding calibration curves.

Additionally, near-infrared spectroscopy (NIRS) was employed to determine the crude protein content in all samples, using an on-demand calibration (Foss DS2500, wheat small cup, Foss Analytics, Hilleroed, Denmark).

### Data Analysis

Secondary structure determination of wheat proteins was performed using Python v3.13.2 in Visual Studio Code 1.101.2 (Microsoft, Redmond, WA, United States) in accordance with the protocol described by Yang et al.^25^ This protocol enables the determination of protein secondary structure by performing spectral deconvolution based on the inverted second-derivative of the amide I band. Hemoglobin and lysozyme were used to verify this protocol (see Table S1). Spectra preprocessing was performed using the SciPy library version 1.15.2.^34^ In summary, baseline correction was performed on four spectra of each wheat protein at a concentration of 10 mg/mL. For all spectra, second-derivative preprocessing was performed with either 7- or 9-point Savitzky–Golay filtering and second-degree polynomial orders using the savgol_filter function of the scipy.signal module. Thereafter, the spectra were truncated in the amide I region (1700–1600 cm^–1^), inverted, followed by zero baseline correction. To assist peak assignment, published deconvoluted amide I band frequencies in H_2_O were used as reference values.^25^ Peak fitting was then performed using the Voigt function from the voigt_profile of the scipy.special module, which represents the convolution of Gaussian and Lorentzian functions. The Levenberg–Marquardt algorithm was performed using the curve_fit function of scipy.optimize module to obtain the best fit, which are the reduced χ^2^ below 10^–6^ and the adjusted *R* ^2^ > 0.99. The secondary structural content of each spectrum was calculated by averaging the percentages of the areas under the bands attributed to each specific secondary structure and the total area of amide I using the trapezoid module in the NumPy library.^35^ The code can be found online.^36^

Outliers in the 720 spectra (60 samples with three replications for each of four protein fractions) were identified using Hotelling and orthogonal distances derived from their projections in the principal component space of each protein fraction, computed in R v4.4.2 within Rstudio 2024.09.1 (Posit, PBC, Boston, MA, United States).^37^ All of the data were found to be free of outliers. Subsequently, all triplicate spectra were averaged to produce a single spectrum for each sample. Spectra preprocessing was performed in the prospectr package.^38^ The Savitzky–Golay gap segment derivatives were calculated using a filter width of 11 and a third-degree polynomial order. The DescTools package was employed to integrate the area under the curve of the spectra.^39^ The amide II band area was integrated using the trapezoid integration method in the range 1590–1480 cm^–1^ along with baseline correction prior to integration. The protein contents (PrC), expressed in g per 100 g of the sample, was calculated from the standard curve as follows:

4 PrC⁡(g100g)=(A‐intercept)×Vs⁡(mL)×100×CFslope×m⁡(mg)

where *A* is the area of the amide II band, *V*_s_ is extractant volume in mL, *m* is sample mass in mg, and CF is a correction factor.

An analysis of normality was performed using the Shapiro–Wilk’s test in Rstudio for each protein fraction and region prior to conducting a deeper analysis. We found that all data were normally distributed (*p* > 0.05; see Table S2). The effects of the regions on protein fractions were analyzed by ANOVA followed by Tukey’s HSD using basic aov then TukeyHSD functions of R.

The ASCA analysis was conducted in the HDANOVA package, and the plots were generated using ggplot2 and ggpubr.^40–42^ ASCA can be used to test the effects on multivariate data, such as MIR spectra.^43^ ASCA was used to understand the effect of the sampling site (*X*_SS_) and variety (*X*_V_) as well as the interaction (*X*_SS×*V*_) using the following equation

5 $$X=X_{\mathrm{SS}}+X_{V}+X_{\mathrm{SS}\times V}+X_{\mathrm{residuals}}$$

where *X* is all MIR protein spectra, unaveraged, and with second derivative preprocessing. The processed spectra were autoscaled using the scale function of R prior to ASCA of the HDANOVA package. The significance of all effects on the MIR spectra was evaluated by permutation testing (*n* = 1000).

## Results and Discussion

Wheat protein fractionation usually employs the Osborne protocol, requiring consecutive extractions.^4,30^ These consecutive extraction steps make the overall process difficult to control. Lyophilization might be a solution to this problem since it involves removing the solvent before combining it with the extraction solvent. However, this process is time and energy consuming. Dupont et al.^31^ developed a method for extracting gliadins using 0.25% SDS in borate buffer. For glutenins extraction, the concentration of SDS was raised to 2% at 60 °C. As a result, avoiding organic solvents, such as 1-propanol or ethanol, the overall extraction procedure becomes easier to handle. The effectiveness of the fractionation procedure was evaluated using SDS–PAGE under reducing conditions (Figure S2), confirming that protein fractions were separated at each extraction step and that the protein calibrants had the same characteristics as the sample extracts. However, comparison of the MIR spectra between gliadins and glutenins, obtained using the method of Schalk et al. for calibrant preparation and Dupont et al. for sample extraction, indicated a significant difference (*p* < 0.05; see Figure S1). Therefore, correction factors were applied prior to quantifying the fractions.

The raw MIR spectra of the four protein fractions, namely, albumins, globulins, gliadins, and glutenins, are presented in Figure 1A–D. Differences in the amide I band (1700–1600 cm^–1^) can be observed between all protein fractions, since this band indicates the secondary structure of the proteins.^23,24^ Smooth bell-shaped curves are shown in the albumins fraction (Figure 1A), whereas globulins (Figure 1B) exhibit a slight decline near their highest peak around 1630 cm^–1^. The gliadins (Figure 1C) spectra display a similar pattern to albumins but with a wavy curvature at 1610 cm^–1^. The broadest amide I band was observed in glutenins (Figure 1D). By applying the second derivative to the spectra, these differences can be enhanced (see Figure S3).

**Figure 1:**
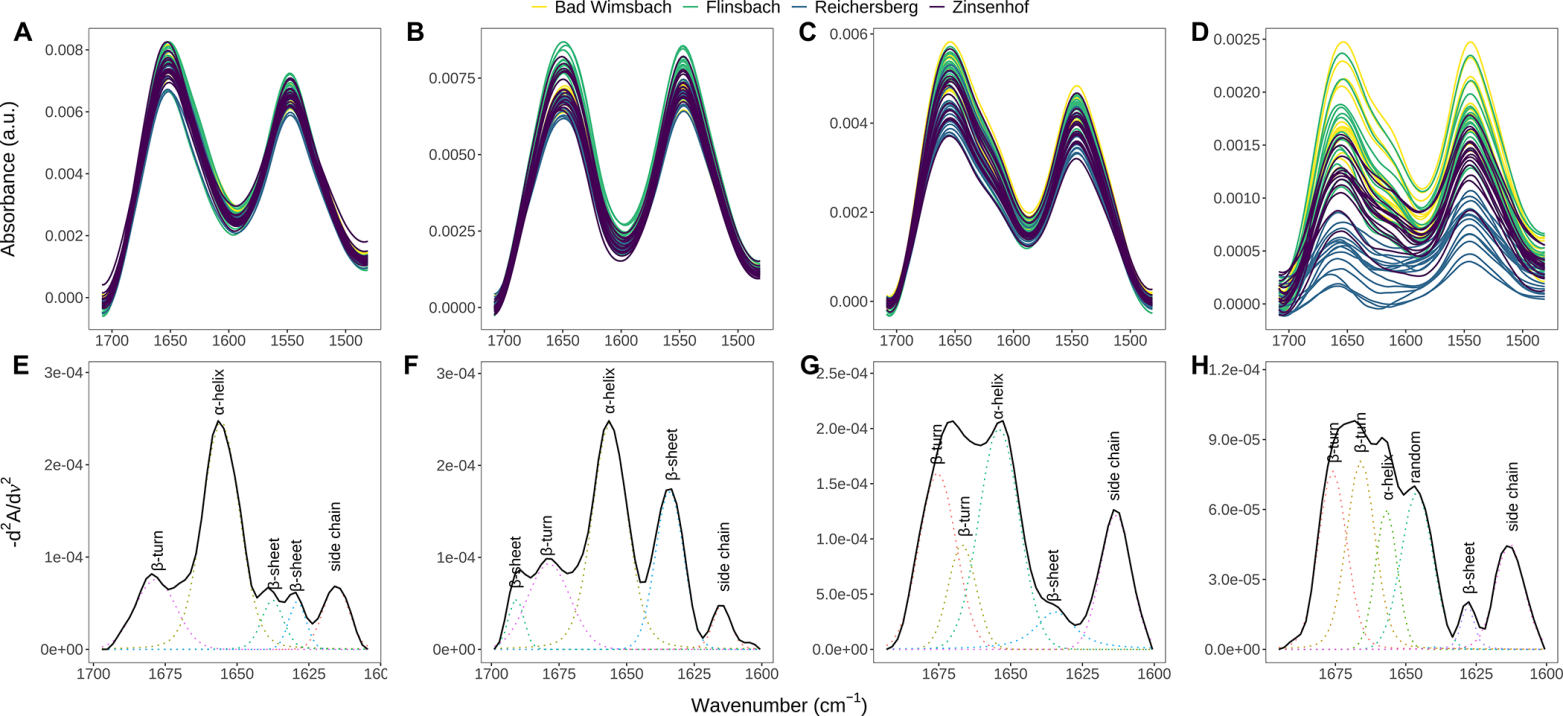
Mid-infrared spectra of wheat protein samples (A–D) and their secondary structures (E–H): albumins (A, E), globulins (B, F), gliadins (C, G), and glutenins (D, H).

Investigating the amide I band through a second-derivative spectral analysis, we found that different secondary structures are present in the protein fractions. The albumins (Figure 1E) consist of 57.8% of α-helix at 1657 cm^–1^, relatively the same amount of β-turn (1679 cm^–1^) and β-sheet (1637 and 1629 cm^–1^) structures around 16%, and 10.1% of side chain. These results are supported by earlier literature^44–46^ that found α-amylase inhibitor and nonspecific lipid transfer proteins (LTP) in wheat, proteins in albumins fraction, mainly consist of α-helix. In globulins (Figure 1F), a 45.9% α-helix (1656 cm^–1^) followed by β-sheet with 31.0%, which is a total of two regions, at 1689 and 1634 cm^–1^ was found. Furthermore, a shoulder corresponding to β-sheet and β-turn in the range 1700–1675 cm^–1^ was also identified.

Gliadins (Figure 1G) and glutenins (Figure 1H) appear to have similar second-derivative spectra; however they are composed of different secondary structures in detail (see Table S3). For gliadins, β-turn and α-helix are the most prominent structures observed, and they share a similar percentage, approximately 37%. This observation is consistent with the findings of past studies,^47–49^ with α- and γ-gliadins having relatively high levels of β-turn and α-helix, while glutenins contain 44.8% of β-turn and 15.3% of α-helix. The dominant structural feature in HMW glutenins is β-turn,^2,50^ whereas the secondary structure of LMW glutenins resembles that of gliadins, contributing to the formation of α-helix.^2,47^ A higher percentage of β-turn and random coils in the glutenin fraction is likely due to the presence of ω-gliadins impurities. A circular dichroism spectroscopy study by Tatham et al.^49^ found that ω-gliadins are rich in β-turn and random coils; this finding is supported by reported high proline content that can induce these secondary structures.^2,51^ In addition, the combination of heating and surfactant in glutenins extraction may have resulted in gradual degradation of the α-helix.^2^ This could explain the lower content of α-helix in our result than Tatham et al.^50^

The side chain peak around 1610 cm^–1^ is attributed to the NH bending vibration in the amide bond of glutamine.^23,52^ The side chain percentages of gliadin, glutenin, albumin, and globulin fractions are 16.7%, 13.3%, 10.1%, and 4.6%, respectively (Table S3), exhibiting a decreasing trend consistent with glutamine content in protein fractions, with respective compositions of 41%, 34%, 22%, and 15%.^2^ Moreover, the higher content of proline also impedes the formation of α-helix and β-sheet structure but induces creating β-turn structure.^51,53^ The content of proline in albumins, globulins, gliadins, and glutenins is 8.9%, 5.0%, 14.3%, and 10.7%, respectively.^2^ This can be linked to our finding of a higher amount of α-helix and β-sheet in albumins and globulins than in gliadins and glutenins. In contrast, the high amount of glutamine in gliadins and glutenins can contribute to the α-helix structure.^53^

As shown in Figure 1A–D, the amide I and II bands exhibit relatively high absorption in all wheat samples, compared to the noise-floor of the FTIR enabling straightforward protein quantification based on the Lambert–Beer law. The amide I band, however, is prone to inference caused by the bending vibration of H_2_O at 1640 cm^–1^.^23,28^ Therefore, the amide II band was chosen for protein quantification (Figure 2A). The evaluation of the working range of all calibration curves is shown in Table 1. We found that all protein fractions meet the Eurachem acceptance criteria for the working range.^33^ The LOQ values of albumins, globulins, gliadins, and glutenins are 0.082, 0.093, 0.029, and 0.246 mg/mL, respectively. In terms of repeatability, all relative standard deviations (RSDs) of protein fractions fall within the acceptance range of the HorRat values for 0.01 mass fraction, which range from 1.2% to 5.2%.^55^ Total protein recovery relative to the Kjeldahl method was 91.1%, consistent with DuPont et al., who reported recoveries of 85–95% depending on the wheat variety.^31^ This excellent performance suggests the workflow holds potential for automated liquid handling using ATR-MIR flow-cells.^56,57^

**Figure 2:**
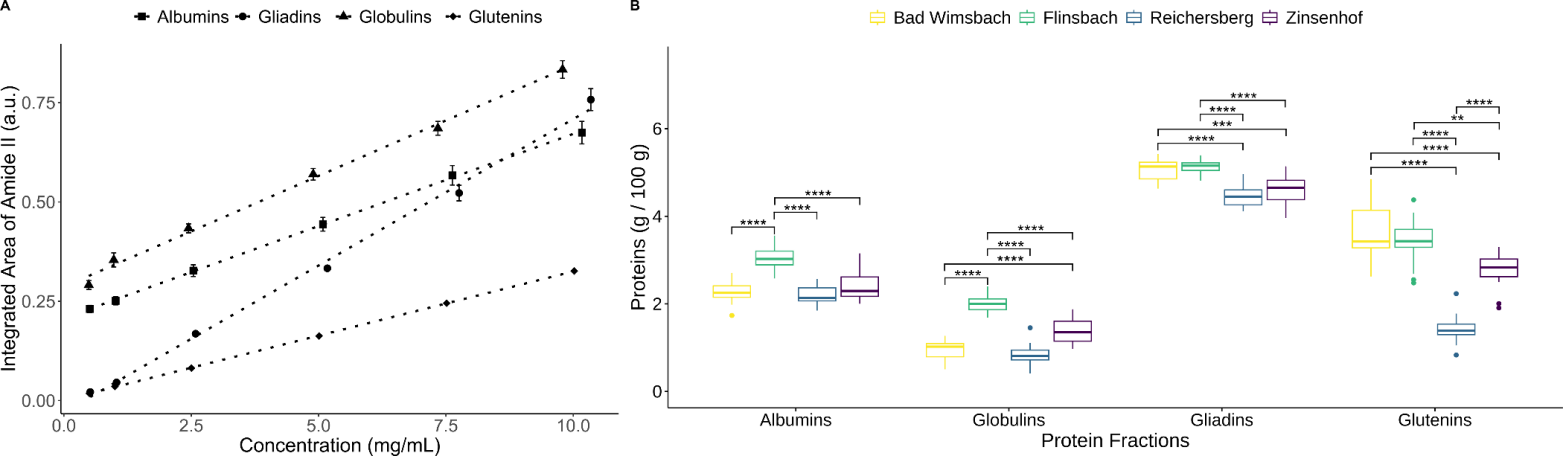
Calibration curves for the protein fractions (A) and comparison of the concentrations of albumins, globulins, gliadins, and glutenins in the four different sampling sites (B).

**Table 1:** Detailed Overview of the Analytical Performance for the Protein Fractions

	albumins	globulins	gliadins	glutenins
slope	0.046	0.056	0.074	0.032
intercept	0.207	0.286	–0.029	0.002
adjusted *R* ^2^	0.9994	0.9941	0.9953	0.9999
LOQ (mg/mL)	0.082	0.093	0.029	0.246
RSD (%)	2.4	2.1	3.0	4.3

To study the effect of the sampling site on the concentration of protein fractions, one-way ANOVA followed by Tukey’s multiple comparison was performed. In Figure 2B, Flinsbach exhibits the highest concentrations of albumins and has a significant difference (*p* < 0.001) from the other regions. In terms of globulins, Zinsenhof and Flinsbach indicate significant protein content differences to others. Bad Wimsbach and Flinsbach contain a higher content of gliadins and glutenins than Reichersberg and Zinsenhof. It is interesting to note the consistency of general patterns of Reichersberg and Zinsenhof in these figures. The individual protein concentrations of the samples are listed in Table S4. Despite an indication of systematic negative bias due to incomplete protein extraction (∼91%), a strong linear correlation was observed between the total protein concentration of our method and the values obtained by NIRS (*R* ^2^ = 0.767; see Figure S4).

The relative protein compositions of all sampling sites are shown in Figure S5. Across all regions except Reichersberg, a consistent pattern is observed where gliadins show the highest percentage values, followed by glutenins and albumins, and globulins with the smallest relative concentrations. Higher content of albumins than glutenins in Reichersberg could be attributed to medium to heavy sandy loam soil conditions in this area (see Table S5). The environmental conditions in Reichersberg might have led the plant to increase the number of proteins in the albumins fraction, which are primarily responsible for regulating, the metabolism, and protective functions.^58,59^

When using ASCA, we found that all protein fractions are significantly affected by all factors (*p* < 0.001; see Table S6). Analyzing the ASCA score plots revealed that the different wheat varieties were not clearly separatable (Figure S6); however, clear clusters associated with sampling sites are observed (Figure S7) for the respective protein fractions. Albumins show a relatively noticeable clustering between sampling sites, although some overlap is still present, which is expected given that albumins concentration are influenced by environmental condition.^58,59^ The other protein fractions display clear separation in only two of four sampling areas: Flinsbach and Zinsenhof in globulins (Figure S7B), Bad Wimsbach and Flinsbach in gliadins (Figure S7C), and Zinsenhof and Reichersberg in glutenins (Figure S7D). Based on this information, all spectra of the four fractions were combined, which led to distinct clusters in the score plot for the effect of the sampling sites (Figure 3A). To gain additional insights into this model, the loading plots on the sampling sites were investigated (Figure 3B). Analysis of the loading plot revealed that the first component (Comp. 1) is strongly influenced by the amide II band, which is associated with the overall protein content, where the highest content of protein fractions is shifted toward the negative side. This finding is in line with the findings presented in Figure 2B, which indicate that the highest and lowest protein fractions are found in the Flinsbach and Reichersberg regions, respectively. For the second component (Comp. 2), albumins and globulins contribute negatively to their Comp. 1 loading, contrary to gliadins and glutenins. An interesting observation is that in albumins fraction, a high negative contribution is shown in 1556–1560 cm^–1^, which could be attributed to the carboxylic group absorption from aspartic and glutamic acid present in side chains of the proteins.^23^ Commonly, the proportions of aspartic and glutamic acids in albumins are 5.8% and 22.6%, respectively.^2^ It is therefore possible that the separation of Bad Wimsbach is caused by a low content of these amino acids in its albumins. This shows that the presented method can be utilized to analyze the secondary structure of wheat protein fractions, to obtain quantitative information, and potentially discriminate samples according to their origin in a straightforward manner.

**Figure 3:**
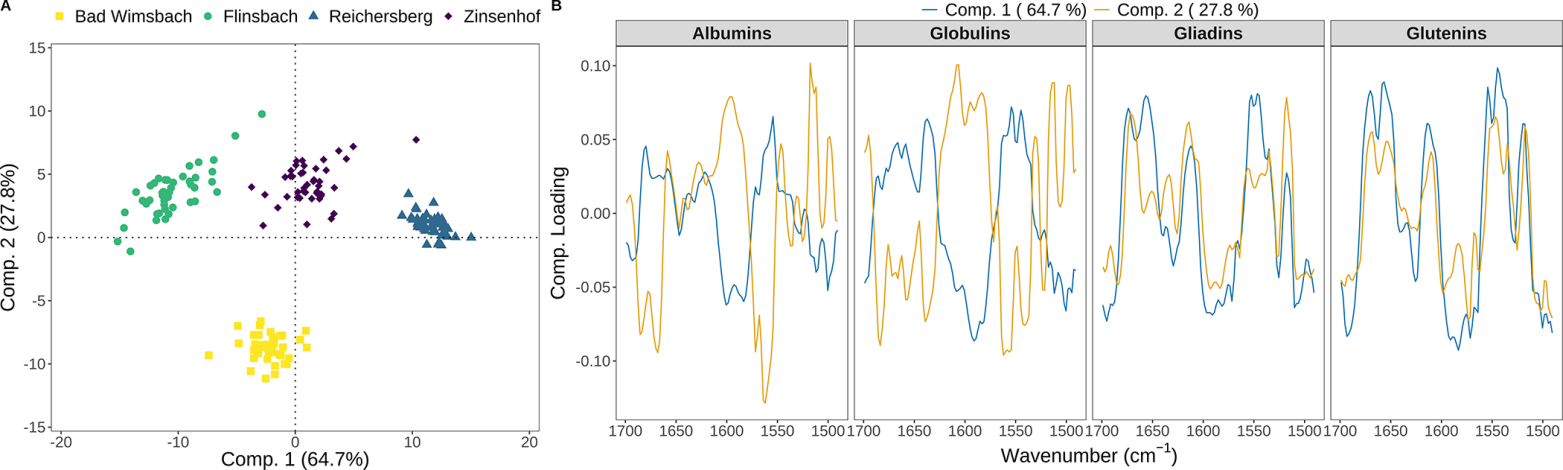
Analysis of variance simultaneous component analysis of the combined second derivative of the protein fractions spectra: score plot for the effect of the sampling sites (A) and the corresponding loading plot (B).

## Conclusions and Outlook

In this study, we demonstrated that ATR-MIR can be used to study wheat proteomes, enabling the analysis of the secondary structure of wheat proteins. The proposed workflow enables the rapid and straightforward analysis of the Osborne protein fractions. In addition to studying the secondary structures of the protein fractions, the potential for discrimination according to sample origin was shown by pairing the Osborne fractionation with ATR-MIR. During method validation, we found that the working range, repeatability, and LOQ indicated excellent performance. Therefore, in the future, the proposed method could be employed prior to more complex proteomic approaches such as LC-MS, to study the effects on different protein fractions, contributing to intolerance research or, after further refinement, to analyze individual protein fractions in greater detail.

## Supplementary Material



## Notes

Tables detailing verification data of the protocol used to determine the protein secondary structure, normal distribution confirmation of the analyzed data, quantitative data of the secondary structure of the wheat protein fractions, quantitative protein data obtained by NIRS and ATR-MIR, soil characteristics of sampling sites, and ASCA results; figures providing spectral comparison between Dupont et al. and Schalk et al. protein extraction methods, SDS–PAGE results, different preprocessed protein spectra, NIRS and ATR-MIR total protein correlation, relative mean composition of wheat protein fractions, and ASCA score and loading plots showing the effects of wheat variety and sampling site (PDF)
