# Supplementary material for: Mid-Infrared Spectroscopy for the Qualitative and Quantitative Analysis of the Wheat Proteome
Source: Anal Chem. 2026 Jan 21;98(4):2775–82. doi: 10.1021/acs.analchem.5c05258 (PMC12874215; doi:10.1021/acs.analchem.5c05258)
Supplement: Supplementary file 1 [file ac5c05258_si_001.pdf]

Supporting Information

Mid-Infrared Spectroscopy for the Qualitative and Quantitative Analysis of the Wheat Proteome

Dedy L. Nadeak,[a] Michael Wiederstein,[a] Sabine Baumgartner,[a] Elisabeth Reiter,[b] Rudolf Krska,[a,c,d] and Stephan Freitag[a]*

[a]Department of Agricultural Sciences, Institute of Bioanalytics and Agro-Metabomics, BOKU University, 3430 Tulln an der Donau, Austria

[b]Austrian Agency for Health and Food Safety GmbH, Institute for Animal Nutrition and Feed, 1220 Vienna, Austria

[c]Institute for Global Food Security, School of Biological Sciences, Queen's University Belfast, BT7 1NN Belfast, UK

[d] FFoQSI GmbH – Austrian Competence Centre for Feed and Food Quality, Safety and Innovation, 3430 Tulln an der Donau, Austria

*corresponding email: stephan.freitag@boku.ac.at

Abstract

This document provides additional data regarding the verification of protein secondary structure determination and extraction protocols, including comparative spectra and SDS-PAGE results between the extraction protocols of Dupont *et al.*[1] and Schalk *et al.*[2] It presents statistical validation metrics, specifically Shapiro-Wilk normality tests conducted prior to subsequent ANOVA and Tukey's test. Detailed results from ANOVA simultaneous component analysis (ASCA) investigating the effects of wheat variety and sampling site are also provided. Furthermore, this document contains soil characteristic data for the sampling sites, graphs showing different pre-processed mid-infrared spectra, and a quantitative comparison between the proposed ATR-MIR protocol and routine NIRS calibration methods.

Table of Contents

Table S1. Result obtained during the verification of the protein secondary structure determination protocol by Yang *et al.*[1] S3

Table S2. *p*-values from the Shapiro-Wilk test indicated that all samples conformed to a normal distribution ($p > 0.05$) S4

Table S3. Results of the secondary structure determination using the protocol of Yang *et al.*[1] S5

Table S4. Protein content of all wheat fractions across 60 wheat samples obtained by the proposed ATR-MIR protocol and the content obtained by a commercially available near-infrared based calibration (FOSS DS2500, wheat small cup). S6

Table S5. Soil characteristics in all sampling sites[3] S8

Table S6. *p*-values and percentage of explained variance obtained by ASCA for the effect of sampling site, variety and their interaction in protein fractions spectra with second derivative pre-processing S9

Figure S1. Comparison between the spectra of gliadins (A) and glutenins (B), which were obtained by the extraction protocol of Dupont *et al.*[7] (yellow) and the method of Schalk *et al.*[8] for calibrant isolation (dark blue). ASCA on the obtained spectra revealed a significant effect of the extraction methods ($p < 0.05$) on the MIR spectra. S10

Figure S2. SDS-PAGE of protein extracts based on Laemmli method.[4] Protein calibrants, namely albumins, globulins, gliadins and glutenins, are in columns 1-4, respectively. Wheat extracts according to Dupont, namely water, 0.5 M NaCl, 0.25% SDS, and 2% SDS soluble, are in columns 5-8, respectively. S11

Figure S3. Raw and pre-processed spectra of all protein fractions. The application of the first and second derivative aimed to reduce baseline drift and improve peak resolution, especially in regions with overlapping protein bands. S12

Figure S4. Correlation between crude protein by NIRS and total proteins by ATR-MIR S13

Figure S5. Relative mean composition ± 2SD of wheat protein fractions, albumins, globulins, gliadins, and glutenins in four different sampling sites S14

Figure S6. ASCA score and loading plot of the second derivative of albumins, globulins, gliadins, and glutenins for the effect of wheat variety ($p < 0.001$). S15

Figure S7. ASCA score and loading plot of the second derivative of albumins, globulins, gliadins, and glutenins for the effect of the sampling sites ($p < 0.001$) S16

References S17

Table S1. Result obtained during the verification of the protein secondary structure determination protocol by Yang *et al.*[3]

Protein	Technique	α-helix (%)	β-sheet (%)	β-turn (%)	Random (%)
**Hemoglobin**	X-ray[a]	87	0	7	8
	Infrared[b]	82.1 ± 2.6	5.0 ± 1.5	11.2 ± 2.0	0
**Lysozyme**	X-ray[a]	45	19	23	13
	Infrared[c]	42.3 ± 3.2	19.1 ± 2.3	17.5 ± 4.6	15.5 ± 6.6

[a] X-ray data were obtained from Levitt and Greer[4] as referenced in the protocol by Yang et.al.[3]
[b] Purchased from Sigma Aldrich, product ID: H2625.
[c] Purchased from Sigma Aldrich, product ID: L6876.

Table S2. *p*-values from the Shapiro-Wilk test indicated that all samples conformed to a normal distribution ($p > 0.05$)

Sampling Sites	Albumins	Globulins	Gliadins	Glutenins
**Bad Wimsbach**	0.951	0.381	0.360	0.688
**Flinsbach**	0.531	0.605	0.889	0.407
**Reichersberg**	0.225	0.537	0.234	0.411
**Zinsenhof**	0.245	0.611	0.991	0.185

Table S3. Results of the secondary structure determination using the protocol of Yang *et al.*[3]

Protein fractions	Wavenumber (cm^{-1})	Assignment	Mean ± 2SD (%)
**Albumins**	1679	β-turn	17.2 ± 2.6
	1657	α-helix	57.8 ± 3.2
	1637, 1629	β-sheet	15.2 ± 4.4
	1615	side chain	10.1 ± 1.9
**Globulins**	1679	β-turn	18.7 ± 2.9
	1656	α-helix	45.9 ± 4.5
	1689, 1634	β-sheet	31.0 ± 3.1
	1615	side chain	4.6 ± 1.4
**Gliadins**	1675, 1666	β-turn	38.3 ± 2.3
	1654	α-helix	36.9 ± 4.2
	1634	β-sheet	7.2 ± 3.7
	1613	side chain	16.7 ± 2.1
**Glutenins**	1675, 1667	β-turn	44.8 ± 4.3
	1657	α-helix	15.3 ± 5.0
	1648	random coil	21.8 ± 0.4
	1690, 1638, 1628	β-sheet	4.3 ± 3.9
	1613	side chain	13.3 ± 0.6

Table S4. Protein content of all wheat fractions across 60 wheat samples obtained by the proposed ATR-MIR protocol and the content obtained by a commercially available near-infrared based calibration (FOSS DS2500, wheat small cup).

Sampling Areas	Albumins (g/ 100 g)	Globulins (g/ 100 g)	Gliadins (g/ 100 g)	Glutenins (g/ 100 g)	Total Proteins (g/ 100 g)	Crude Protein by NIRS (g/ 100 g)
**Bad Wimsbach**	2.7 ± 0.1	0.7 ± 0.0	4.9 ± 0.1	4.3 ± 0.4	12.5 ± 0.5	12.4
	1.9 ± 0.0	0.9 ± 0.0	5.3 ± 0.3	3.4 ± 0.2	11.5 ± 0.4	12.5
	2.2 ± 0.1	0.8 ± 0.0	4.8 ± 0.1	3.3 ± 0.2	11.2 ± 0.2	12.7
	2.5 ± 0.2	1.1 ± 0.1	4.6 ± 0.2	4.5 ± 0.2	12.7 ± 0.3	12.5
	2.0 ± 0.1	1.1 ± 0.0	5.3 ± 0.1	3.3 ± 0.0	11.6 ± 0.1	11.8
	2.2 ± 0.0	1.3 ± 0.1	5.3 ± 0.3	4.8 ± 0.3	13.6 ± 0.4	13.4
	2.3 ± 0.1	1.0 ± 0.0	5.4 ± 0.1	3.0 ± 0.1	11.8 ± 0.2	12.2
	2.1 ± 0.1	1.0 ± 0.1	5.1 ± 0.6	3.5 ± 0.4	11.7 ± 0.7	14.3
	2.2 ± 0.2	0.5 ± 0.0	5.2 ± 0.3	4.0 ± 0.3	12.0 ± 0.4	12.0
	2.4 ± 0.1	1.3 ± 0.0	4.7 ± 0.3	2.7 ± 0.4	11.2 ± 0.5	12.2
	2.6 ± 0.1	0.8 ± 0.1	5.2 ± 0.3	3.7 ± 0.2	12.3 ± 0.3	13.0
	2.3 ± 0.2	0.6 ± 0.0	5.2 ± 0.1	2.6 ± 0.2	10.7 ± 0.3	11.9
	1.7 ± 0.1	1.0 ± 0.1	5.0 ± 0.2	3.4 ± 0.1	11.2 ± 0.2	13.0
	2.2 ± 0.1	1.3 ± 0.1	4.8 ± 0.9	4.3 ± 0.4	12.5 ± 1.0	11.3
	2.2 ± 0.1	1.1 ± 0.1	4.9 ± 0.2	3.3 ± 0.2	11.5 ± 0.4	12.4
**Flinsbach**	3.3 ± 0.3	2.1 ± 0.0	5.1 ± 0.4	2.7 ± 0.2	13.2 ± 0.6	13.9
	3.3 ± 0.1	2.4 ± 0.1	5.2 ± 0.1	3.7 ± 0.3	14.5 ± 0.4	14.4
	3.5 ± 0.2	2.4 ± 0.1	5.2 ± 0.2	3.7 ± 0.4	14.9 ± 0.6	14.4
	2.5 ± 0.1	1.8 ± 0.0	5.1 ± 0.6	2.5 ± 0.3	11.9 ± 0.6	13.5
	2.5 ± 0.1	2.0 ± 0.0	4.8 ± 0.3	3.4 ± 0.0	12.8 ± 0.3	14.3
	2.9 ± 0.1	1.9 ± 0.1	5.3 ± 0.5	3.3 ± 0.4	13.4 ± 0.7	12.8
	2.6 ± 0.3	1.9 ± 0.0	5.1 ± 0.3	3.3 ± 0.3	12.9 ± 0.5	13.1
	3.0 ± 0.2	2.0 ± 0.0	5.3 ± 0.2	3.4 ± 0.2	13.7 ± 0.3	12.7
	3.0 ± 0.2	2.0 ± 0.1	5.3 ± 0.2	4.1 ± 0.5	14.5 ± 0.5	13.4
	2.8 ± 0.2	1.8 ± 0.1	5.0 ± 0.9	4.4 ± 0.3	13.9 ± 1.0	14.9
	2.9 ± 0.1	2.2 ± 0.1	5.4 ± 0.4	3.8 ± 0.5	14.4 ± 0.6	14.5
	2.9 ± 0.1	2.0 ± 0.0	4.9 ± 0.6	2.6 ± 0.3	12.4 ± 0.7	14.5
	3.0 ± 0.2	2.1 ± 0.1	5.0 ± 0.5	3.5 ± 0.7	13.6 ± 0.9	13.9
	3.3 ± 0.1	1.7 ± 0.0	5.2 ± 0.3	3.3 ± 0.6	13.5 ± 0.7	13.1
	3.0 ± 0.2	2.3 ± 0.1	5.2 ± 0.2	3.5 ± 0.3	14.0 ± 0.4	14.5
**Reichersberg**	2.5 ± 0.1	0.8 ± 0.1	4.6 ± 0.4	1.3 ± 0.1	9.3 ± 0.4	8.8
	2.0 ± 0.1	1.0 ± 0.1	5.0 ± 0.0	1.6 ± 0.1	9.6 ± 0.1	9
	1.8 ± 0.1	1.0 ± 0.0	4.3 ± 0.1	1.3 ± 0.1	8.4 ± 0.2	7.5
	2.0 ± 0.0	1.0 ± 0.1	4.8 ± 0.1	1.4 ± 0.1	9.2 ± 0.1	10.2
	2.0 ± 0.2	0.9 ± 0.0	4.9 ± 0.1	2.2 ± 0.0	10.1 ± 0.2	8.2
	2.5 ± 0.2	0.4 ± 0.0	4.6 ± 0.4	1.8 ± 0.3	9.3 ± 0.6	9.4
	2.2 ± 0.1	0.8 ± 0.0	4.5 ± 0.3	1.6 ± 0.0	9.0 ± 0.4	10.3
	2.3 ± 0.1	0.8 ± 0.0	4.4 ± 0.4	1.5 ± 0.1	9.0 ± 0.5	9.4

Sampling Areas	Albumins (g/ 100 g)	Globulins (g/ 100 g)	Gliadins (g/ 100 g)	Glutenins (g/ 100 g)	Total Proteins (g/ 100 g)	Crude Protein by NIRS (g/ 100 g)
	2.5 ± 0.1	0.9 ± 0.1	4.3 ± 0.3	1.1 ± 0.0	8.8 ± 0.4	8.8
	2.1 ± 0.1	1.5 ± 0.1	4.4 ± 0.0	0.8 ± 0.0	8.8 ± 0.2	9.3
	2.1 ± 0.3	0.8 ± 0.0	4.2 ± 0.0	1.4 ± 0.1	8.5 ± 0.3	8.1
	2.4 ± 0.1	0.5 ± 0.0	4.1 ± 0.1	1.4 ± 0.0	8.4 ± 0.2	8.5
	2.0 ± 0.0	1.1 ± 0.1	4.6 ± 0.3	1.1 ± 0.1	8.8 ± 0.4	8.3
	2.2 ± 0.1	0.7 ± 0.0	4.3 ± 0.1	1.5 ± 0.2	8.7 ± 0.2	8.7
	2.1 ± 0.0	0.6 ± 0.1	4.2 ± 0.3	1.4 ± 0.0	8.2 ± 0.3	8.3
**Zinsenhof**	2.5 ± 0.1	1.1 ± 0.0	4.8 ± 0.2	3.3 ± 0.3	11.7 ± 0.4	12.1
	2.1 ± 0.1	1.0 ± 0.0	4.3 ± 0.4	3.2 ± 0.4	10.5 ± 0.6	10.8
	2.1 ± 0.1	1.3 ± 0.1	4.5 ± 0.7	2.6 ± 0.1	10.5 ± 0.7	12
	2.3 ± 0.1	1.4 ± 0.1	4.9 ± 0.6	2.6 ± 0.2	11.2 ± 0.6	11.8
	2.0 ± 0.1	1.4 ± 0.0	4.3 ± 0.5	3.1 ± 0.4	10.8 ± 0.7	10.3
	2.5 ± 0.1	1.8 ± 0.1	4.6 ± 0.0	2.7 ± 0.1	11.6 ± 0.1	10.3
	2.4 ± 0.1	1.7 ± 0.2	4.8 ± 0.3	2.9 ± 0.3	11.9 ± 0.5	11.1
	2.2 ± 0.0	1.1 ± 0.0	4.4 ± 0.5	2.9 ± 0.2	10.6 ± 0.5	11.4
	2.2 ± 0.2	1.1 ± 0.0	4.8 ± 0.0	3.1 ± 0.3	11.2 ± 0.4	10.6
	2.6 ± 0.1	1.8 ± 0.0	5.1 ± 0.3	1.9 ± 0.1	11.5 ± 0.4	11.9
	2.2 ± 0.0	1.5 ± 0.0	4.0 ± 0.0	3.0 ± 0.5	10.7 ± 0.5	12
	2.0 ± 0.2	1.2 ± 0.0	4.3 ± 0.5	2.0 ± 0.2	9.6 ± 0.6	11.1
	3.1 ± 0.2	1.9 ± 0.1	4.7 ± 0.1	2.7 ± 0.3	12.4 ± 0.4	10.7
	2.6 ± 0.3	1.6 ± 0.1	4.7 ± 0.1	2.5 ± 0.5	11.4 ± 0.6	10.1
	2.9 ± 0.2	1.4 ± 0.0	5.0 ± 0.4	2.8 ± 0.2	12.2 ± 0.5	11.2

Table S5. Soil characteristics in all sampling sites[5]

Sampling Sites	Soil Characteristics
Bad Wimsbach	district of Wels; 393 m / 899 mm / 10.0 °C.<br>Pseudovergleyte Lockersediment-Braunerde brown earth, deep, medium humus with a slightly acidic to neutral soil reaction. (Pseudovergleyte Lockersediment-Braunerde refers to a brown soil developed from loose sediments, with seasonal water stagnation in the upper layers.)
Flinsbach	district of St. Pölten-Land; 312 m / 685 mm / 10.4 °C.<br>Brown earth, partly gleyed, deep, medium to heavy.
Reichersberg	district of Ried im Innkreis; 350 m / 827 mm / 9.7 °C.<br>Deep, medium-heavy sandy loam soils. Little water-permeable subsoil leads to occasional surface waterlogging, pH value 5.3 to 6.8, humus content 1.5 to 2.8%.
Zinsenhof	district Melk, Lower Austria; Central European transitional climate with Atlantic influence, 260 m / 704 mm / 10.3 °C.<br>Gleyed brown earth from fine alluvial material, sandy loam to loamy silt, well supplied with water, medium to deep, carbonate-containing, slightly acidic to neutral, humic.

Table S6. *p*-values and percentage of explained variance obtained by ASCA for the effect of sampling site, variety and their interaction in protein fractions spectra with second derivative pre-processing.

Protein Fractions	Sampling Sites (%)*	Wheat Variety (%)*	Sampling Site x Wheat Variety (%)*
Albumins	15.3	17.9	43.4
Globulins	24.6	13.9	38.0
Gliadins	19.9	14.2	38.8
Glutenins	29.6	15.3	32.5
All fractions combined	22.3	15.3	38.2

*p-value < 0.001

Figure S1. Comparison between the spectra of gliadins (A) and glutenins (B), which were obtained by the extraction protocol of Dupont *et al.*[1] (yellow) and the method of Schalk *et al.*[2] for calibrant isolation (dark blue). ASCA on the obtained spectra revealed a significant effect of the extraction methods ($p < 0.05$) on the MIR spectra.

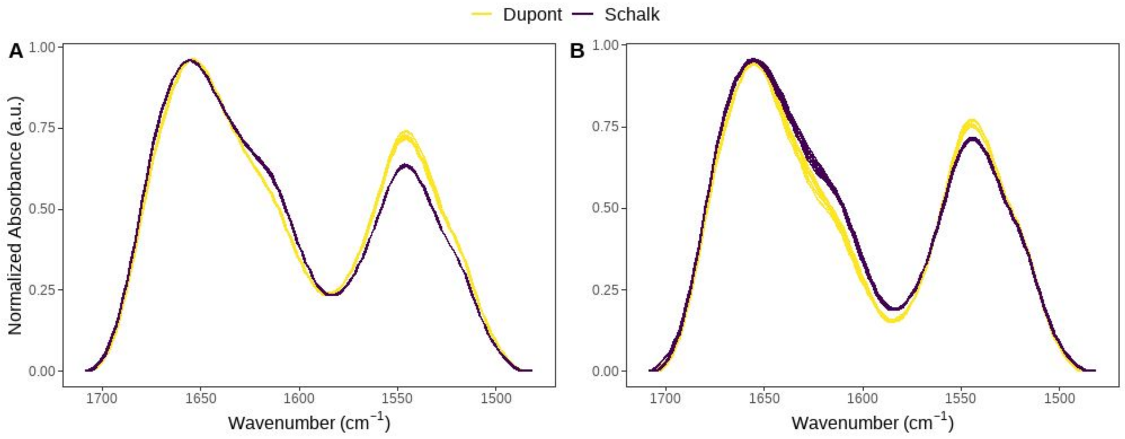

Figure S2. SDS-PAGE of protein extracts based on Laemmli method.[6] Protein calibrants, namely albumins, globulins, gliadins and glutenins, are in columns 1-4, respectively. Wheat extracts according to Dupont, namely water, 0.5 M NaCl, 0.25% SDS, and 2% SDS soluble, are in columns 5-8, respectively.

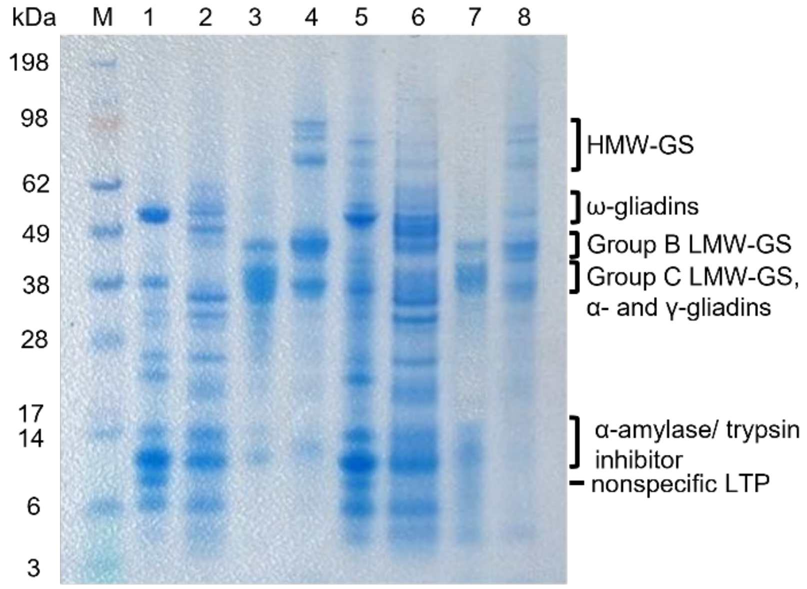

The effectiveness of the fractionation procedure was evaluated by SDS-PAGE technique under reducing condition. As shown in **Figure S2**, the first four samples represent the in-house produced wheat protein calibrants, while the remaining columns represent protein fractions obtained from extraction. A similar pattern can be observed between albumins (column **1**) and water-soluble proteins (column **5**), as well as between globulins (column **2**) and salt-soluble proteins (column **6**). Both protein fractions, albumins and globulins, have a similar range of molecular weight (MW), 14–62 kDa, which is consistent with the findings of Singh and Skerritt.[7] In the albumins, MW 9 and 12-16 kDa are considered to be nonspecific lipid transfer proteins (LTP) and *alpha*-amylase/ trypsin inhibitors, respectively. While globulins within the range of MW 30 and 43 kDa are identified as globulin and serpin, respectively.[8]

In column **3**, the subtypes of gliadins, namely α- and γ-gliadins, are displayed with MW of 30-40 kDa, which have similar patterns with the 0.25% SDS-soluble proteins (column **7**). In terms of glutenins and its subtypes, high molecular weight (HMW) and low molecular weight (LMW) glutenins, are observed in the range 95-97 kDa and 30-45 kDa, respectively. Both glutenins subtypes are displayed in column **4** (glutenins calibrant) and column **8** (2% SDS-soluble proteins). This result, however, also reveals a small amount of LMW glutenins in the 0.25% SDS extract (column **7**) and ω-gliadins (66 kDa) in the 2% SDS extract (column **8**), which is in line with the findings of Dupont *et al.*[1] As a result, SDS-PAGE confirmed that the protein fractions were separated in every extraction step, and the protein calibrants had similar characteristics to the extracts.

Figure S3. Raw and pre-processed spectra of all protein fractions. The application of the first and second derivative aimed to reduce baseline drift and improve peak resolution, especially in regions with overlapping protein bands.

1. Albumins

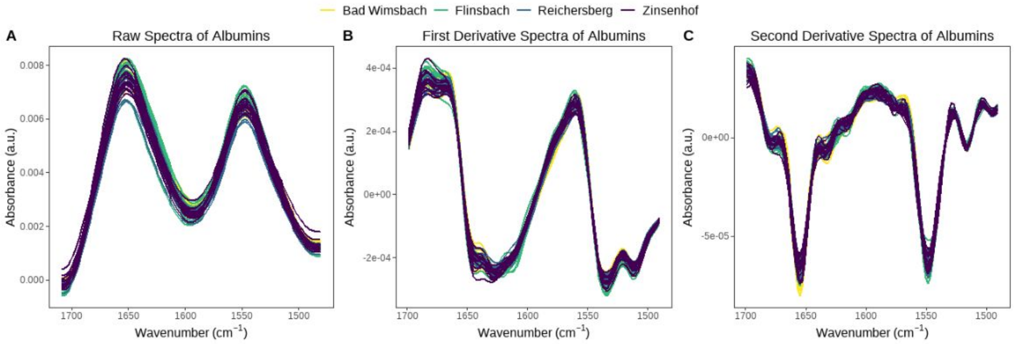

2. Globulins

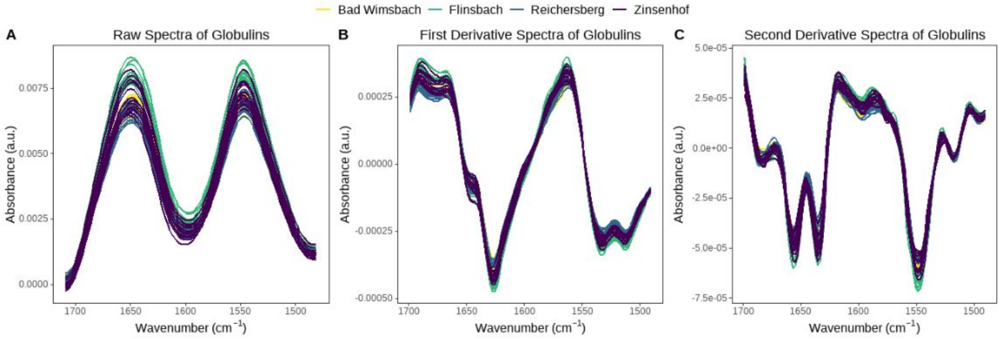

3. Gliadins

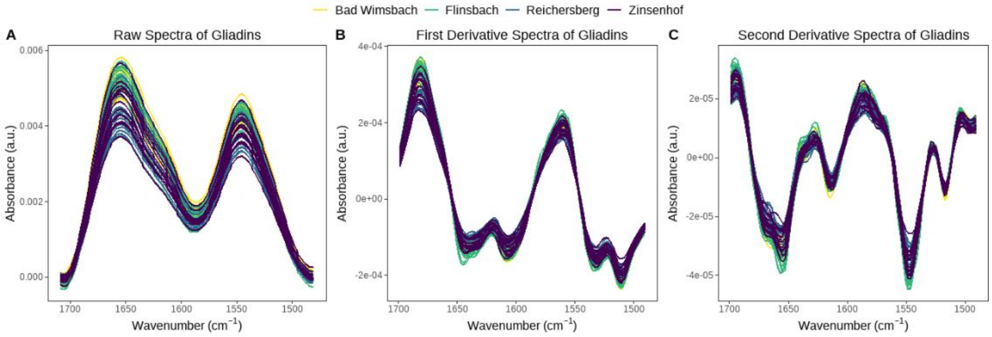

4. Glutenins

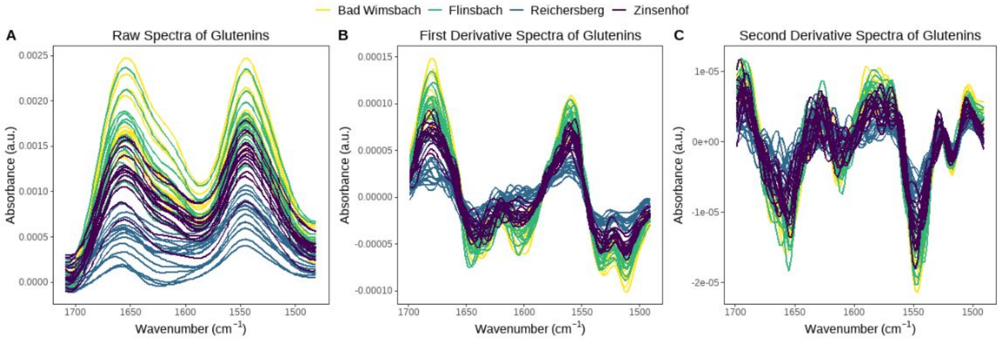

Figure S4. Correlation between crude protein by NIRS and total proteins by ATR-MIR

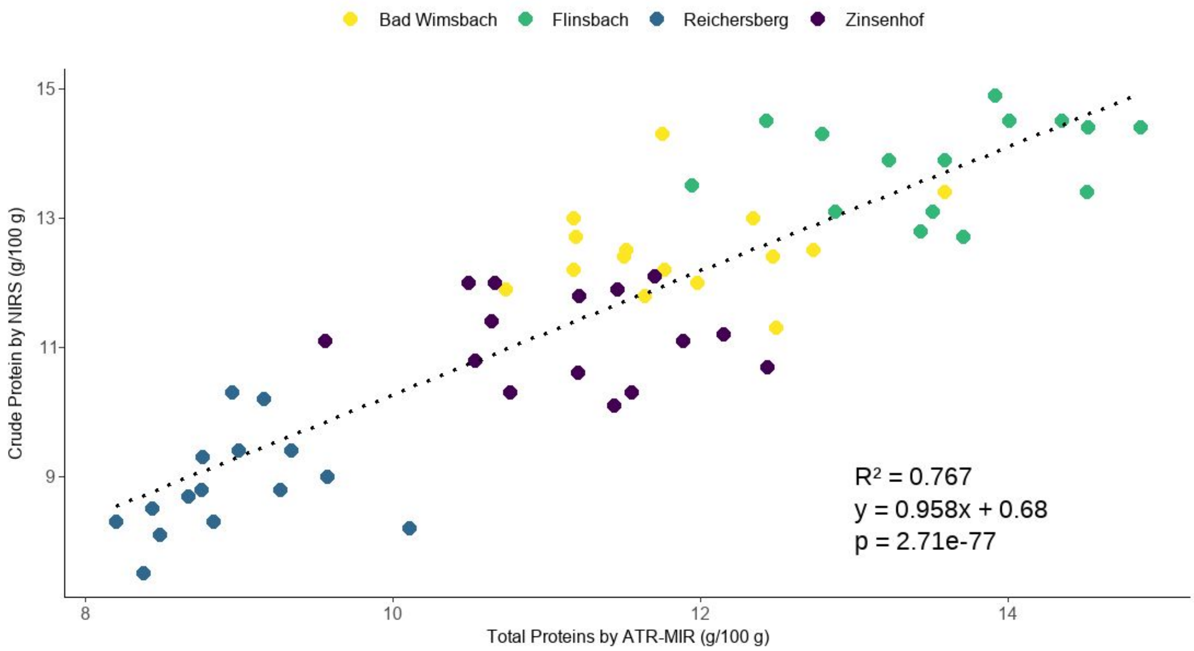

Figure S5. Relative mean composition ± 2SD of wheat protein fractions, albumins, globulins, gliadins, and glutenins in four different sampling sites

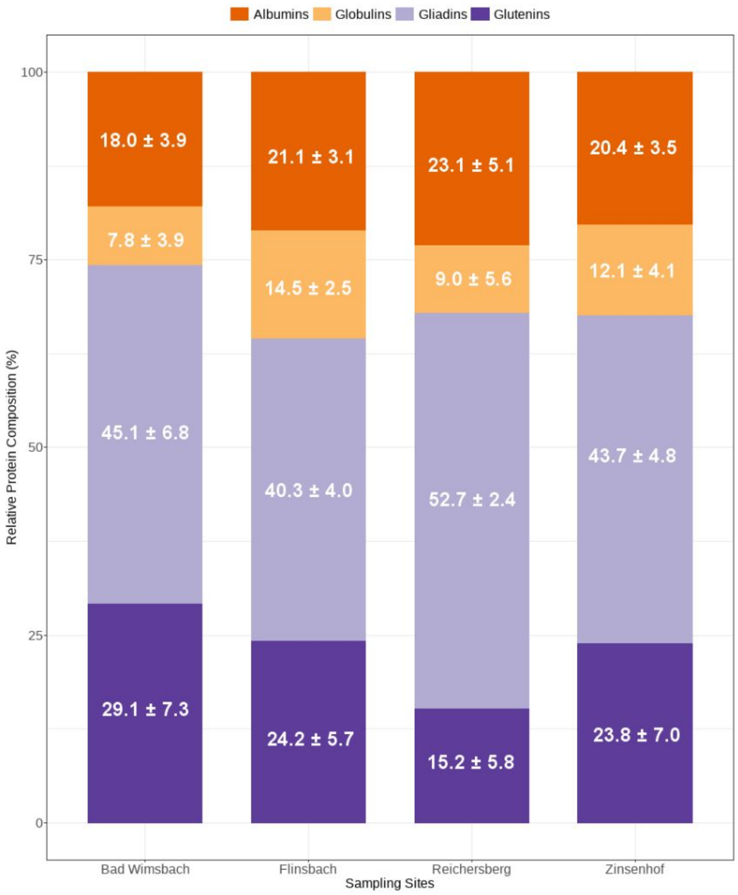

Figure S6. ASCA score and loading plot of the second derivative of albumins, globulins, gliadins, and glutenins for the effect of wheat variety ($p < 0.001$).

A. Albumins

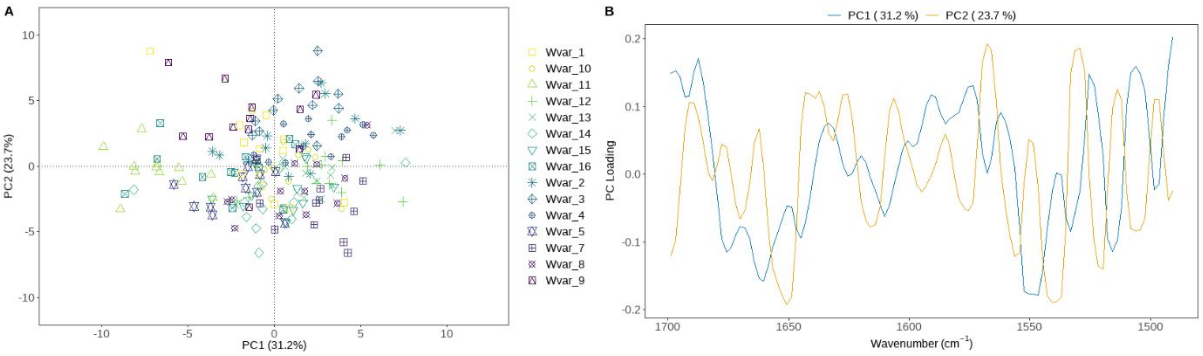

B. Globulins

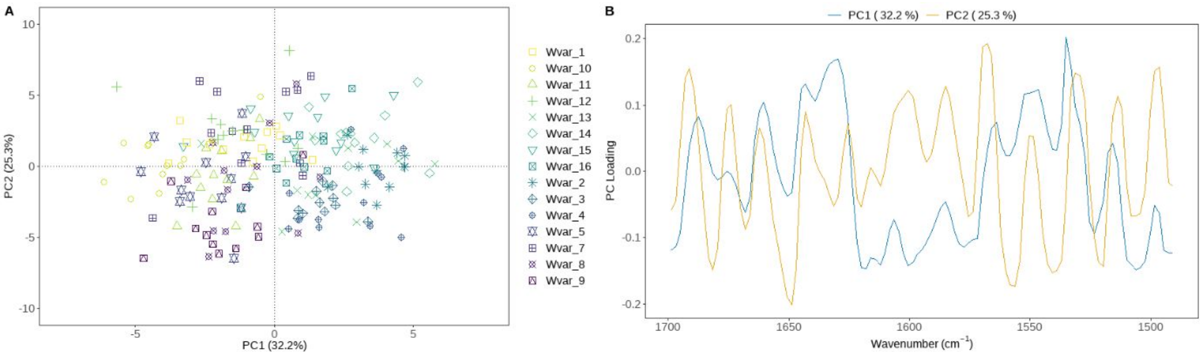

C. Gliadins

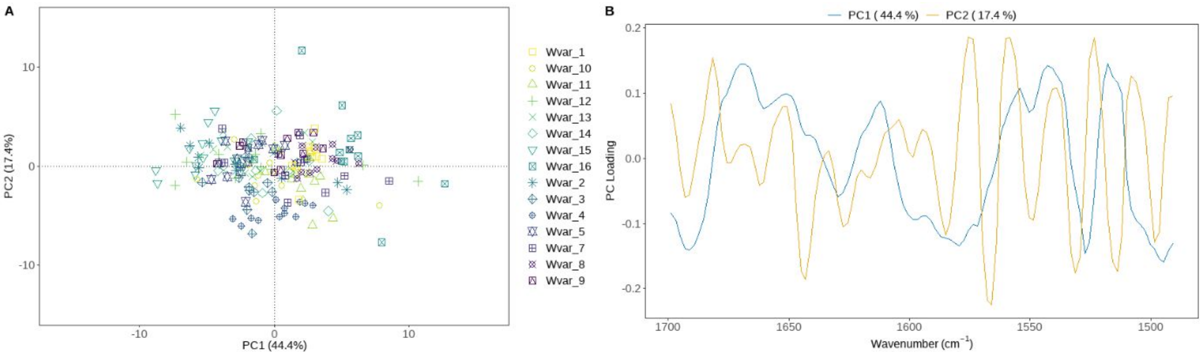

D. Glutenins

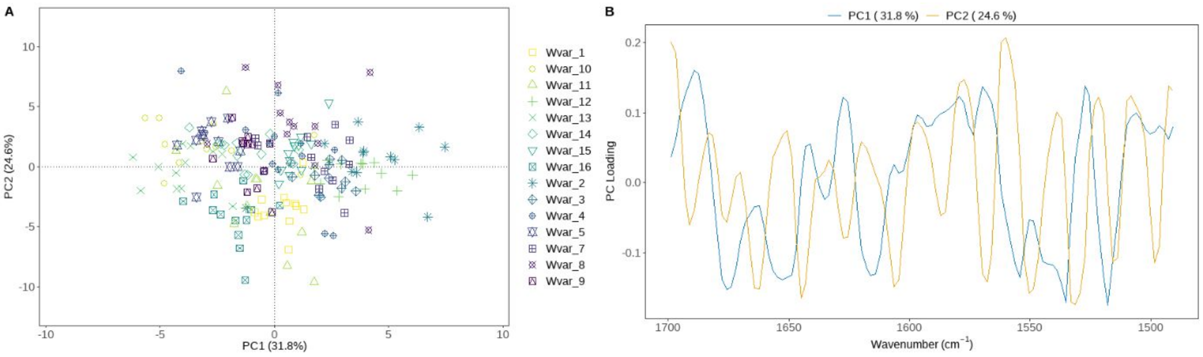

Figure S7. ASCA score and loading plot of the second derivative of albumins, globulins, gliadins, and glutenins for the effect of the sampling sites ($p < 0.001$).

A. Albumins

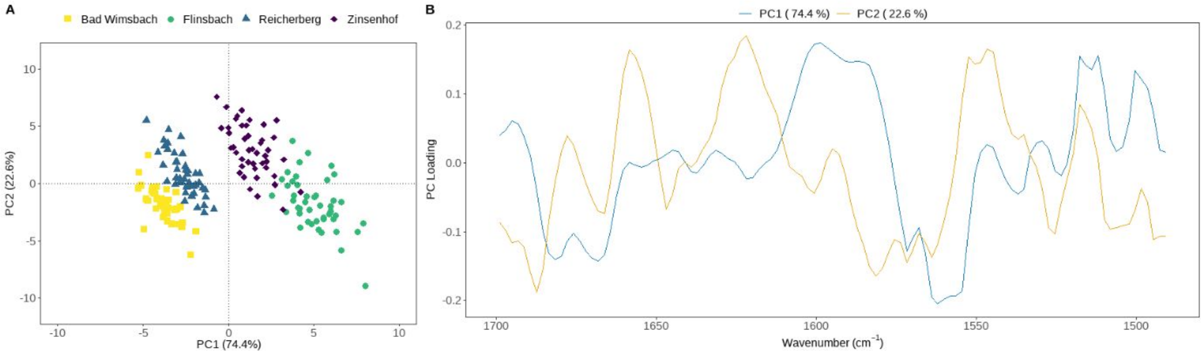

B. Globulins

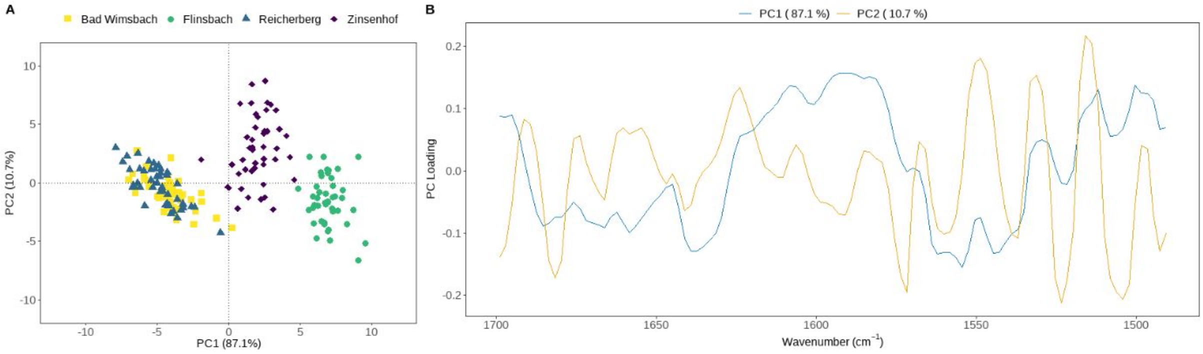

C. Gliadins

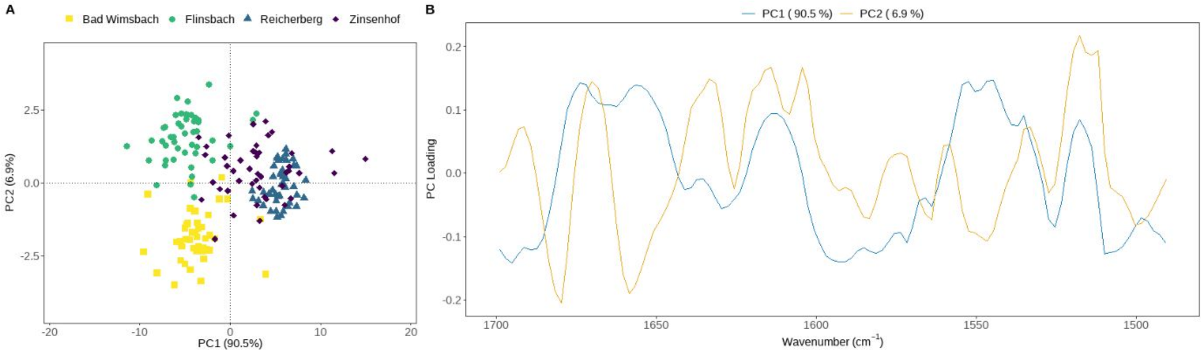

D. Glutenins

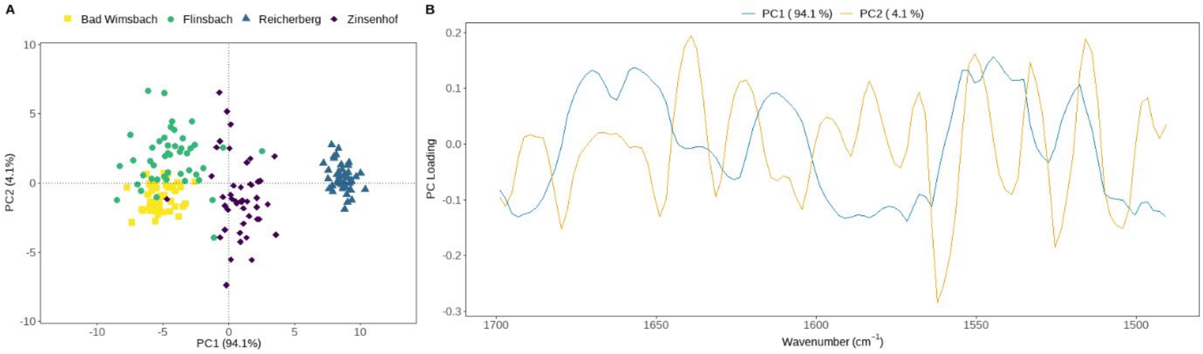

References

(1) DuPont, F. M.; Samoil, V.; Chan, R. Extraction of up to 95% of Wheat (*Triticum Aestivum*) Flour Protein Using Warm Sodium Dodecyl Sulfate (SDS) without Reduction or Sonication. *J. Agric. Food Chem.* **2008**, *56* (16), 7431–7438. https://doi.org/10.1021/jf800776b.

(2) Schalk, K.; Lexhaller, B.; Koehler, P.; Scherf, K. A. Isolation and Characterization of Gluten Protein Types from Wheat, Rye, Barley and Oats for Use as Reference Materials. *PLOS ONE* **2017**, *12* (2), e0172819. https://doi.org/10.1371/journal.pone.0172819.

(3) Yang, H.; Yang, S.; Kong, J.; Dong, A.; Yu, S. Obtaining Information about Protein Secondary Structures in Aqueous Solution Using Fourier Transform IR Spectroscopy. *Nat Protoc* **2015**, *10* (3), 382–396. https://doi.org/10.1038/nprot.2015.024.

(4) Levitt, M.; Greer, J. Automatic Identification of Secondary Structure in Globular Proteins. *Journal of Molecular Biology* **1977**, *114* (2), 181–239. https://doi.org/10.1016/0022-2836(77)90207-8.

(5) AGES. *Österreichische Beschreibende Sortenliste 2023 Landwirtschaftliche Pflanzenarten*; ISSN: 1560-635X.https://bsl.baes.gv.at/; 21/2023; Republik Österreich, 2023.

(6) Laemmli, U. K. Cleavage of Structural Proteins during the Assembly of the Head of Bacteriophage T4. *Nature* **1970**, *227* (5259), 680–685. https://doi.org/10.1038/227680a0.

(7) Singh, J.; Skerritt, J. H. Chromosomal Control of Albumins and Globulins in Wheat Grain Assessed Using Different Fractionation Procedures. *Journal of Cereal Science* **2001**, *33* (2), 163–181. https://doi.org/10.1006/jcrs.2000.0351.

(8) Pastorello, E. A.; Farioli, L.; Conti, A.; Pravettoni, V.; Bonomi, S.; Iametti, S.; Fortunato, D.; Scibilia, J.; Bindslev-Jensen, C.; Ballmer-Weber, B.; Robino, A. M.; Ortolani, C. Wheat IgE-Mediated Food Allergy in European Patients: α-Amylase Inhibitors, Lipid Transfer Proteins and Low-Molecular-Weight Glutenins. *Int Arch Allergy Immunol* **2007**, *144* (1), 10–22. https://doi.org/10.1159/000102609.
